# An Intratumoral Aneurysm and an Extrarenal Synchronous Cystic Tumour in a Case of a Renal Cell Carcinoma

**DOI:** 10.1155/2021/8878429

**Published:** 2021-04-06

**Authors:** Supun De Silva, Lalani De Silva, Susantha De Silva, Priyani Amarathunga

**Affiliations:** ^1^Postgraduate Institute of Medicine, University of Colombo, Sri Lanka; ^2^Urology Unit 02, National Hospital of Sri Lanka, Colombo, Sri Lanka; ^3^Department of Pathology, Faculty of Medicine, University of Colombo, Sri Lanka

## Abstract

**Background:**

Renal cell carcinoma is a heterogeneous group of malignant tumors originating from the kidney. We report a case of a renal cell carcinoma with two very rare associates, i.e., a large intratumoral aneurysm and a synchronous extrarenal cystic tumor outside the main tumor. *Case Presentation*. A 31-year-old woman, who presented with painless hematuria and loin pain, was diagnosed to have a large renal mass measuring 15 × 9 × 8.5 cm with an intralesional arterial aneurysm measuring 4.5 × 3.5 cm on radiological examination. During surgery, a separate cystic tumor measuring 5 × 4.5 × 4 cm with distinct vascular supply was noted anteromedial to the kidney, in addition to the renal mass. The histology of the main tumor was compatible with t(6:11) type microphthalmia-associated transcription factor (MiT) family translocation RCC. The aneurysm was of venous origin histologically, and a radiologically demonstrable arteriovenous fistula was recognized retrospectively. The extrarenal cyst has also showed similar histology to that of main tumor and had no evidence of a degenerated lymph node. *Discussion*. Although few cases were reported with various vascular anomalies associated with a renal tumor, this is the first ever case to find an arteriovenous fistula with a secondary venous aneurysm located inside a malignant renal mass. Similarly, no solid RCC is reported to present with an extrarenal malignant cystic nodule. The prognostic and oncological significance of the extrarenal malignant cyst is unclear. Both of these extraordinary features of this case were not properly identified on preoperative imaging. Reviewing the preoperative imaging when pathology reports are available helps to overcome difficulties in making the final diagnosis of complex cases.

**Conclusion:**

RCCs can house vascular anomalies like arteriovenous fistula and venous aneurysms and can exist with concomitant extrarenal malignant cystic nodules.

## 1. Introduction

Renal cell carcinoma (RCC) is a heterogeneous group of malignant tumors originating from the kidney. It is known to be associated with a variety of vascular anomalies. Several types of vascular anomalies like renal artery aneurysms (RAAs), venous aneurysms (VAs), and arteriovenous fistulas (AVF) were found to coexist with RCC. However, all of those vascular anomalies reported so far were located outside the tumor mass or in most cases, outside the kidney. Multifocality and coexistence of both solid and cystic components in one tumor are also known phenomena with RCC. In most instances, both of these special features were found inside the ipsilateral kidney. A well-demonstrable extrarenal malignant cystic nodule is never reported to coexist with any type of solid RCC. Here, we report the first ever case to have an intratumoral AVF and a VA together with an extrarenal synchronous malignant cyst in an adult microphthalmia-associated transcription factor (MiT) family translocation RCC. The discovery of two extraordinary features in an extremely rare type of RCC makes this case unique.

## 2. Case Presentation

**Figure 1:**
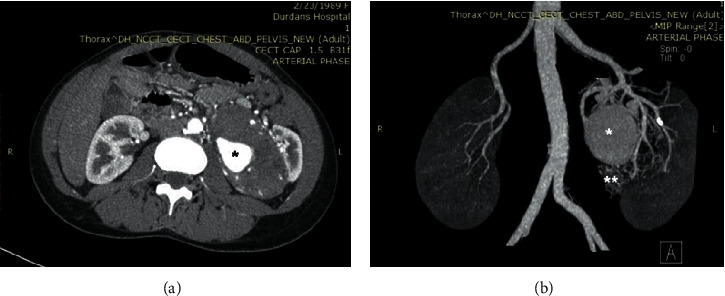
Contrast enhanced CT images of the tumour. (a) An axial section showing the left renal tumour showing the aneurysm (asterisk). The tumour involves the entire kidney except a thin rim of normally enhancing renal tissue at the upper outer region. (b) CT renal angiogram showing multiple feeding arteries and the aneurysm (asterisk). The vasculature of the extrarenal cystic nodule (double asterisks) is also seen inferior to the aneurysm.

A 31-year-old lactating mother was investigated for painless hematuria and vague loin pain. She has given birth to her first baby 10 months before, and by that time, she did not have any symptoms related to the genitourinary system. Her antenatal ultrasound scans also had not detected any evidence of a maternal renal mass. There was no history of any familial cancer syndromes. There was a large, nontender, ballotable mass in the left loin. Apart from that, her physical examination was unremarkable. An ultrasound scan of the abdomen has found a large mass lesion involving the left kidney. A contrast-enhanced computerized tomogram (CECT) further detailed the lesion. It was a large, lobulated, well-demarcated, solid, and cystic tumor measuring 15 × 9 × 8.5 cm arising from the mid to upper zones of the left kidney. Additionally, there was an aneurysm of 4.5 × 3.5 cm inside the tumor, the feeding artery of which could also be identified (Figure 1). There were few suspicious hilar and para-aortic lymph nodes. There was no evidence of distant metastasis and the other kidney was normal. Her serum creatinine was 0.78 mmol/l.

**Figure 2:**
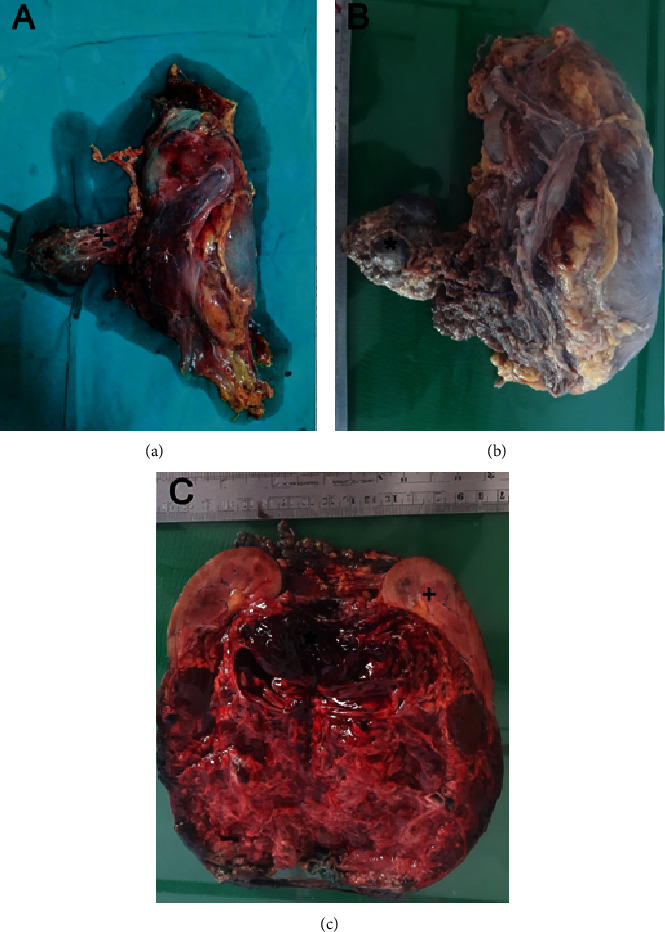
Macroscopic appearance of the tumour. (a) An immediate postoperative image of the specimen showing the left kidney, extrarenal tumor nodule (asterisk) and the vessels supplying it (+). (b) The external appearance of the specimen after formalin fixation demonstrating the separate tumor nodule (asterisk). (c) Cut surface showing the tumour involving the entire kidney sparing a thin rim of normal kidney tissue (+) and the aneurysm filled with a blood clot (asterisk).

She was offered open radicle nephrectomy. During surgery, it was noted that there was another separate soft, cystic lesion anteromedial to the main tumour. It measured 5 × 4.5 × 4 cm and had distinct blood supply (Figure 2). There were multiple hilar and para-aortic lymph nodes, which were sampled. The postoperative period was uneventful, and the patient was discharged on the third postoperative day.

**Figure 3:**
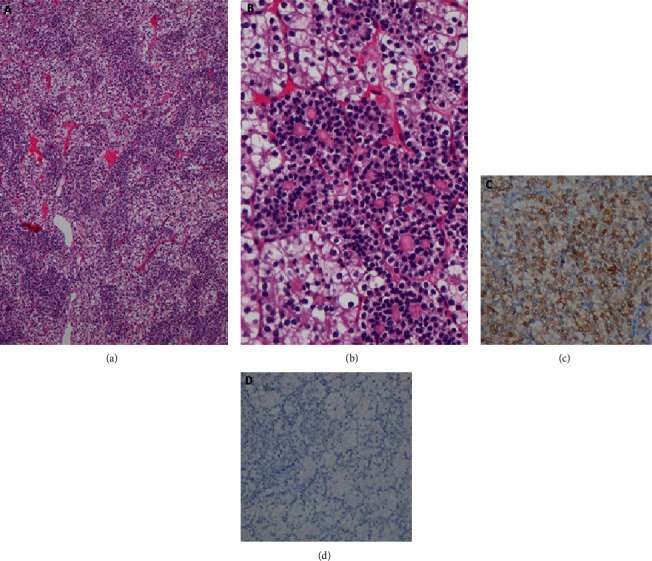
Microscopic appearance of the tumour. (a) Closely packed nests comprising a dual population of cells—H&E 40x. (b) High-power view showing nests of large polygonal cells with clear cytoplasm, surrounded by a thin capillary network. The admixed second population of small cells are forming rosettes around deeply eosinophilic basement membrane like material—H&E 400x. (c) Positive immune-staining for Melan-A. (d) Negative staining for Vimentin.

**Figure 4:**
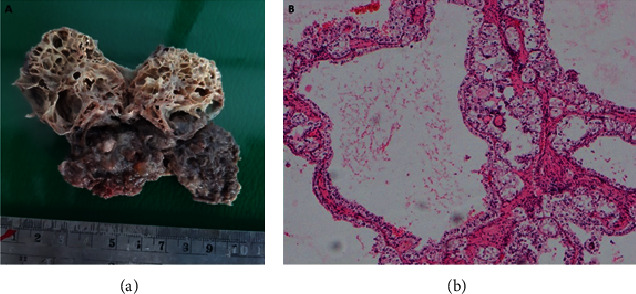
The extrarenal tumor nodule. (a) A cut section showing multilocular cystic structure with thin fibrous septae. (b) Microscopy showing thin-walled cysts lined by a dual population of cells, similar to the main tumour—H&E 200x.

Macroscopically, the main tumor measured 13.5 × 7.5 × 5.5 cm and involved the upper and mid zones of the kidney. It was lobulated and well defined with large cystic and hemorrhagic areas. There was a blood-filled cavity in the posteromedial aspect of the kidney measuring 4.5 × 3.8 × 3.5 cm which is compatible with the radiologically identified aneurysm (Figure 2). The extrarenal cystic lesion was multiloculated with thin septae and was filled with straw color fluid. Microscopically, the tumor was composed of two different populations of malignant cells arranged in nests. The center of the nests was formed by closely packed small rounded cells with central rounded nuclei and scanty eosinophilic cytoplasm. The periphery of the nests had large polygonal cells with central rounded nuclei and abundant clear cytoplasm (Figure 3). Some areas had sheets of large polygonal cells. Numerous psammoma bodies were also found. Immunohistochemical analysis revealed diffuse cytoplasmic positivity for Melan-A and negative staining for Vimentin, HMB 45, and CD 117. All these features were compatible with MiT family translocation RCC. The tumor had invaded the branches of the renal vein and renal sinus fat, but there was no breach of the renal capsule (pT_3a_). The intratumoral cystic lesion was lined by thin-walled vascular endothelium compatible with a venous aneurysm. The septae of the extrarenal cystic nodule were lined by multiple layers of large polygonal cells which were morphologically identical to those in the main tumor. The immunohistochemical staining pattern of it was also identical that of the primary tumour, i.e., diffuse cytoplasmic positivity for Melan-A and negative staining for Vimentin, HMB 45, and CD 117 (Figure 4). Thirteen out of 15 lymph nodes were positive for tumor deposits (pN1). Thus, the final pathological stage of the tumor was pT_3a_, N_1_, and M_0_. A positron emission tomography (PET) scan carried out three months after the surgery did not detect any residual or metastatic tumor in this patient.

## 3. Discussion

Vascular anomalies are not uncommon in highly vascular tumors like RCCs with a higher tendency for vascular invasion and hematogenous spread. Proper identification of the location and the origin of a vascular lesion like an AVF or an aneurysm is pivotal for preoperative planning to avoid catastrophes during surgery. In the index case, the intratumoral aneurysm which was readily identified in the early arterial phase of the CECT was reported as an arterial aneurysm of one of the posterior sectoral branches of the renal artery. However, the wall of the aneurysm was composed of venous tissue histologically. Upon further inquiry into the CT angiography of the lesion, a small venous tributary was also identified in relation to the aneurysm. Therefore, the lesion was finally labeled as a venous aneurysm, distal to an arteriovenous fistula (AVF). An identical case of a venous aneurysm secondary to an AVF was reported by *Ferrante* et al. which was also identified as an arterial aneurysm, on preoperative radiology [1]. Five cases of RAA were reported so far to coexist with a RCC [2–5]. In all of the above cases, the aneurysm was found to be located outside the tumour. This is the only reported case so far, to have an aneurysm located inside a tumour mass of a RCC.

The existence of cystic RCC and coexistence of renal cysts with solid RCC are well known. In this patient, a separate cystic lesion was identified with readily identifiable distinct vascular supply. This lesion, which was retrospectively identified in the CECT, was not recognized as a separate mass in the initial report. The radiology team has considered it as a necrotic area of the main tumor, initially. The discovery of the same malignant epithelium with similar immunostaining pattern in the cyst made the case more interesting. The impact of this malignant nodule upon the staging and the prognosis of this patient cannot be determined as no such case is ever reported. It cannot be considered as a different malignancy because it shares the same histological and immunohistochemical features of the main tumor. It can neither be considered as a lymph node invaded by the tumor as no lymphatic tissue was identified inside the cyst. The origin of this malignant nodule remains elusive.

MiT family translocation renal cell carcinomas are a rare form of RCC having either Xp11 or t(6:11) chromosomal translocations. They are predominantly seen in the pediatric age group but extremely rare in adults and indicate poorer prognosis [6–8]. Xp11 subtype is commoner and found to account for 1.6% of adult RCCs in one series [9]. The t(6:11) translocation carcinomas are much rarer than Xp11 subtype and the incidence of which is 0.02% of all RCC [10]. Morphologically, they tend to mimic the various types of RCCs, including clear cell, papillary, and even epitheloid angiomyolipomas [6]. They are characteristically biphasic, with small cells clustered around basement membrane material and large polygonal epithelioid cells in the periphery which is compatible with the histology of this case report. The histopathological features of the two types are known to overlap, which can explain the abundance of psammoma bodies in the index case, which is a feature of Xp11 RCCs. Cytogenetic and specific immunohistochemical studies (TFE3 and TFEB) are necessary for both the confirmation of MiT translocation and for subtyping. We had to resort to surrogate immunohistochemical markers in this case as those studies are not available in Sri Lanka.

Both extraordinary features of this case were not precisely recognized preoperatively. The final diagnosis of both features necessitated several meetings with the pathology, radiology, oncology, and urology teams. Both the AVF/VA and the separate cystic tumor could be identified retrospectively in CECT. This emphasizes the importance of having multidisciplinary team meetings to discuss complex cases like the index case. Moreover, the oncological and prognostic implications of the extrarenal malignant cyst are a matter for further discussion.

## 4. Conclusion

RCCs can harbor vascular anomalies like AVF and VA which can make surgery challenging. Synchronous malignant cysts can exist even outside the kidney in cases with RCC. Reviewing preoperative imaging when pathology reports are available helps to overcome difficulties in making the final diagnosis in complex cases.

## Data Availability

All data pertaining to this article are available with the authors.
